# Traditional Arabic & Islamic Medicine: A Conceptual Model for Clinicians and Researchers

**DOI:** 10.5539/gjhs.v4n3p164

**Published:** 2012-05-01

**Authors:** Sara Al-Rawi, Michael D Fetters

**Affiliations:** 1 Department of Family Medicine, University of Michigan, Ann Arbor, U.S.

**Keywords:** indigenous health, islamic medicine, tibb nabawi, traditional medicine, health of minorities, folk healing practices

## Abstract

Eighty percent of the population in the developing world relies on traditional medicine, and 70-80% of the population in developed countries utilizes complementary therapies. Though a vibrant healing tradition pervades modern life in the Arab and Muslim world, no clear definition or model exists to organize it’s multiple and intertwined elements. We define Traditional Arabic and Islamic Medicine (TAIM) as a system of healing practiced since antiquity in the Arab world within the context of religious influences of Islam and comprised of medicinal herbs, dietary practices, mind-body therapy, spiritual healing and applied therapy whereby many of these elements reflect an enduring interconnectivity between Islamic medical and prophetic influences as well as regional healing practices emerging from specific geographical and cultural origins. Our definition and conceptual model represents a novel addition to the literature on Arab and Muslim health practices, and presents an opportunity to address a global health concern.

## 1. Introduction

An estimated 80% of the population in much of the developing world relies on traditional systems of medicine, and 70-80% of the population in developed countries have used some form of alternative or complementary medicine (WHO, 2008). Herbal treatments are the most commonly utilized form of traditional medicine, and are lucrative in the international marketplace yielding $5 billion US in revenues in Western Europe, $14 billion US in China and $160 million US in Brazil (WHO, 2008).

The World Health Organization (2008) defines traditional medicine as “the sum total of knowledge, skills and practices based on the theories, beliefs and experiences indigenous to different cultures that are used to maintain health, as well as to prevent, diagnose, improve or treat physical and mental illnesses”. Two examples of widely known traditional systems of medicine include Traditional Chinese Medicine (TCM) and Ayurvedic medicine. Another vibrant and expansive system of healing traditions thrives and pervades modern life in the Arab and Muslim world. Idioms used to connote these healing traditions include Graeco-Arabic or Unani medicine, Islamic Medicine, and Tibb Nabawi or medicine of the Prophet. While sometimes used interchangeably, these traditions each developed in historically distinct times and feature differences in theory and practice. Though discrete features exist within these traditions, they share overarching practices, terminology and historical linkages. Despite an extant, though finite, literature on the various practices of these healing traditions, no clear and concise model exists to distinguish and organise the multiple, intertwined elements.

## 2. Background and Significance

### 2.1 Current Status of TAIM

Azaizeh et al. (Azaizeh, Saad, Cooper, & Said, 2010) recently proposed the term Traditional Arabic & Islamic Medicine (TAIM). Conceptually, this encompassing term recognizes traditional Arabic and Islamic medicine as one system to embrace the entirety of the historical roots and breadth of practices, and represents an innovative step forward. Unfortunately, the authors neither provide a definition nor a conceptual model to describe the proposed term. To advance clinical and academic applications of this healing tradition, a single framework uniting the inter-related and overlapping terminology is necessary. Consequently, our purpose is to provide a working definition of TAIM, and to present a conceptual framework to delineate the scope of TAIM.

### 2.2 Development of Arabic Medicine

The development of Arab medicine occurred in three phases (Saad, Azaizeh, & Said, 2005). The first phase, in the 8^th^ century, entailed the translation of medical works of Hippocrates and Galen, philosophical works by Plato and Aristotle, and mathematical works of Euclid and Archimedes into Arabic (Saad et al., 2005). Hospitals and medical schools flourished in the Arab world, and several Muslim scholars reached a stature in medical sciences that exceeded that of their predecessors (Saad et al., 2005). Of these notable scholars, Rhazes (Al Razi, 846-930) and Avicenna (Ibn Sina, 980-1037), were instrumental in commemorating this period as the Golden Age. The final phase of the development of Arab medicine started in the 12^th^ century when European scholars studied Arab works and translated them into Latin (Saad et al., 2005). The most noteworthy example is the translation of Avicenna’s ‘Canon of Medicine’ in addition to Rhazes book ‘The Comprehensive’, which continued to dominate medical teachings in Europe until the 16^th^ century (Saad et al., 2005).

### 2.3 Origins of TAIM

Traditional Arabic Medicine is the culmination of Graeco-Roman, Chinese, Persian and Ayurvedic theories and practices (Oumeish, 1998). Origins of Islamic medicine can be traced back to the beginning of the Islamic civilization in the 7th century when Islamic scholars and physicians expanded earlier medical sciences with their own discoveries (Oumeish, 1998), and amplified preexisting theoretical principles of medicine into a comprevenhsive system of medicine (Bhikha, 2007).

Table 1 highlights the similarities and differences between the four medical systems, particularly with regards to understanding the etiology of disease, the pathological processes underlying disease, and treatment application in the context of the worldview associated with each. (Bhikha, 2007)

**Table 1:** 

Criteria	Ayurvedic Medicine	Traditional Chinese Medicine	Greek Medicine	Islamic Medicine
Inherent wisdom responsible for health restoration and preservation	Prana	Chi Energy	Physis (healing power of nature)	Physis (healing power of nature)
Active force that maintains equilibrium	Doshas (energy dominance)	Yin and Yang (energy dominance)	Humours (metabolic dominance)	Humours and Tempermental imbalance
Spiritual Influence	Hinduism/Buddhism	Taoism/Confuciansim/Buddism	Abrahamic scriptures	Abrahamic scriptures

### 2.4 Defining TAIM

Formalized theoretical frameworks of traditional healing systems, such as traditional Chinese medicine (TCM) and Ayurveda, as well as Complementary and Alternative Medicine (CAM) in developed countries (NCCAM, 2008a), incorporate manipulative and massage techniques, herbal medicine, dietary practices, meditation, and exercise (Nestler, 2002). This taxonomy provides a useful structure for characterizing TAIM and organizing its elements.

**Figure 1:**
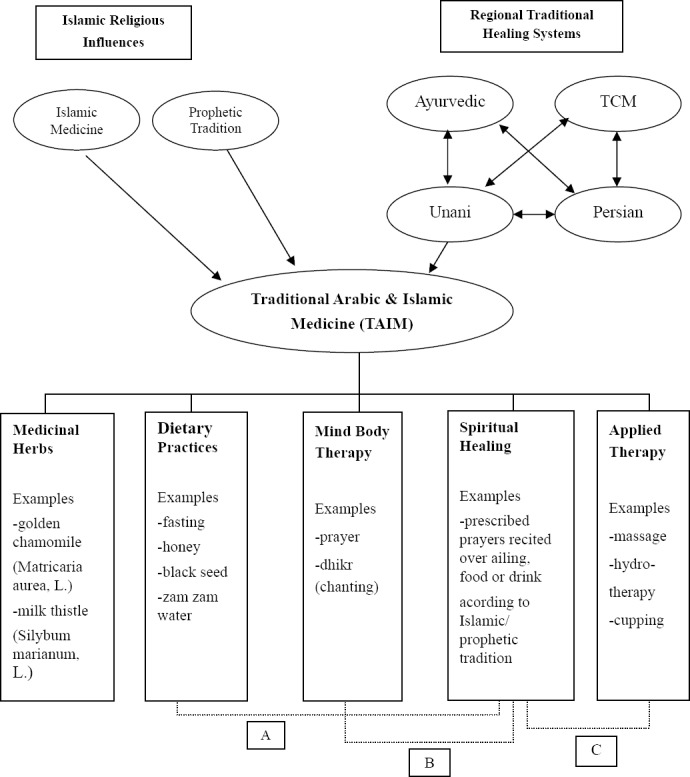
A Unifying Conceptual Model of Traditional Arabic & Islamic Medicine (TAIM) *A, Dietary practices derived from Islamic/prophetic tradition include prescription for fasting and drinking Zam zam water*. *B, Mind-body therapy practices originating from Islamic/prophetic tradition include prayer*. *C, Applied therapy consequential of Islamic/prophetic tradition include cupping*.

Thus, we define Traditional Arabic and Islamic Medicine as a system of healing practiced since antiquity in the Arab world within the context of religious influences of Islam and to be comprised of medicinal herbs, dietary practices, mind-body practices, spiritual healing and applied therapy whereby many of these elements reflect an enduring interconnectivity between Islamic medical and prophetic influences as well as regional healing practices emerging from specific geographical and cultural origins. Below we organise these elements into a unified TAIM conceptual model (Figure 1) and include an illustration of ways that Islamic medical and prophetic influences, as well as regional healing traditions interact together to help define the TAIM model and its underlying five elements.

## 3. Components of TAIM Conceptual Model

### 3.1 Medicinal Herbs

There are approximately 250 plant species currently used in traditional Arabic medicine for the treatment of various diseases (Saad et al., 2005). Medicinal plants are used in the form of herbal teas, syrups, infusions and ointments (Saad et al., 2005). One commonly used herb, *Nigella sativa* (L.), also known as black seed, is traditionally used both as an herb and oil, and is used for the prevention and cure of many ailments in the Middle East and South East Asia. (Al-Ghamdi, 2001) The black seed is also revered in prophetic tradition for its healing ability (Illustration_1). Indications for black seed include respiratory health, immune system support, and stomach and intestinal health (Sharma, Ahirwar, Jhade, & Gupta, 2009).

### 3.2 Dietary Practices

Dietary Practices include the utilization of certain foods, such as honey, (Nagamia, 2003) for its traditional and prophetic indications (Illustraction_2). Honey’s medicinal use spans a wide array of concerns including promoting circulation, stomach and intestinal pain and colic, and as a topical antibiotic (Oumeish, 1999). Other dietary practices include observing a fast, considered the oldest form of natural healing (Chishti, 1991). Observed fasts are in tune with the cycles of the moon, planets and other natural phenomena (Chishti, 1991). Fasting is a core tenant of the Islamic faith, and the primary fast is called Ramadan. The word Ramadan does not mean “fast”; the actual term for fasting is *siyam*, whose root word means “to be at rest” (Chishti, 1991). In addition to the obligatory fast of Ramadan, there are various optional fasts that occur yearly, monthly and some weekly. The methodology of fasting entails abstaining from food, drink, and sexual intercourse for a specified duration of time. When these bodily functions are given the opportunity to rest, they become rejuvenated (Chishti, 1991). While there are physical benefits to fasting, the greatest kind of fasting is that of the mind where a great emphasis is placed on keeping silent except being in divine rememberance. This fast entails restraining the eyes from immoral sight, and the tongue from obscenity, slander and hyprocrisy. While fasting can be a natural practice for maintaining physical health, it also carries immense spiritual rewards as the desired effects occur in the realm of the soul and its evolution (Chishti, 1991).

### 3.3 Mind-body Therapy

Mind-body Therapy denotes techniques designed to enhance the mind’s positive impact on the body, and include such practices as prayer (NCCAM, 2008b). Islamic ritual prayer is both an external and internal meditative practice with a set of physical postures, similar to yoga *asanas*, each with a different meaning and effect both physically and psychologically (Chishti, 1991). Another meditative practice is *Dhikr*, or divine remembrance. Similar to meditation, Dhikr is the process of “listening within, the activation of a presence capable of witnessing inner and outer events without becoming absorbed in them” (Helminski, 2000). The spiritual practice of Dhikr goes beyond attaining clarity and relaxation, however, and becomes about establishing a relationship with the divine (Helminski, 2000). The methodology of Dhikr is generally divided into recollection with the tongue (loud Dhikr), and recollection in the heart (silent Dhikr) (Schimmel, 1975). Considered food for the heart, and medicine for the soul, Dhikr entails repetition of divine names or religious formulae. With the repetition of a rhythmical formula accompanied by music and certain movements, loud Dhikr can be a means of bringing a larger group into an ecstatic state (Schimmel, 1975). In its developed form, Dhikr is usually connected with varying forms of breath control (Schimmel, 1975). This example of meditation, using concentration with breath control, can be seen in other traditions such as the Jesus prayer of early Christian monks, *Nembutsu* within Japanese Buddhism, or *Japa* in Hinduism (Geels, 1996).

### 3.4 Spiritual Healing

In the Islamic tradition, healers rely on both physical and spiritual means to cure disease and promote wellness. Such techniques include recitations, devotions and supplications. For example, a healer will use the recitation of certain prayers together with “breathing upon the sick”, as a prescribed “formula” for healing (Al-Krenawi & Graham, 2000). Prayers are also recited over food and/or water by an alledged saint or sage, and later consumed by the ailing for healing purposes. *Zamzam* water, from a well located in Mecca, the holiest place in Islam, is also consumed for its healing benefits (Abel-Motey, 1997; Al-Enzi & Khan, 2007).

Other types of spiritual healing entail the manipulation of energy patterns within and around the human body (Mirahmadi & Mirahmadi, 2005). Spiritual healers describe the flow of divine energy within the body as “vortices made up of a number of smaller spirals of energy” known as *lata’if* (Mirahmadi & Mirahmadi, 2005). These nine points are also related to the *chakras* of Kundalini Yoga, a component of both Hindu and Buddhist mysticism and to the nodes of the Tree of Life, a core concept in Jewish Kabalistic spirituality (Mirahmadi & Mirahmadi, 2005). Seven of the nine *lata’if* are particularly important in spiritual healing, and illness occurs when one or more of these points are unbalanced as they are considered to be vital focal points of equilibrium within the body (Mirahmadi & Mirahmadi, 2005). Each of these vortices has a different anatomical location, is associated with a different colour of energy, and has varying effects on specific illnesses (Mirahmadi & Mirahmadi, 2005).

## 4. Applied Therapy

Applied therapy includes such traditional methods as massage, hydrotherapy and cupping. Used by various cultures around the world, cupping entails using a glass cup and attaching it to the surface of the skin through a negative pressure created by heat or suction (Akhtar & Siddiqui, 2006). Cupping draws blood, fluids, and energy to the surface, and thus its benefits can be attributed to increasing circulation, and promoting the elimination of toxins stored in the tissues (Akhtar & Siddiqui, 2006). A type of cupping, *hijama* or wet cupping, is taken from prophetic tradition with specified methodology (Akhtar & Siddiqui, 2006). This is a method whereby blood is drawn by vacuum from a small skin incision for therapeutic purposes. Cupping is applied on different parts of the body, and varying forms of cupping are used depending on the nature of disease. Traditional cupping is used to treat up to 72 diseases including toothaches, boils, gout, and elephantiasis (Oumeish, 1998).

The frequent mention of certain practices in the Quran and prophetic tradition, such as the use of black seed (*Nigella sativa*, L.) and honey illustrates the interconnectivity between the conceptual elements and exemplifies the need to include the spiritual and regional practices in the overall model. This is attributable to the traditional medicinal use of black seed and honey worldwide, as well as its regard in Islamic tradition as possessing immense healing qualities.

## 5. Clinical Implications

Several healthcare system implications exist for understanding the role of TAIM among Arab and Muslim patients. Patient’s not discussing its use leads to risks of drug-herb or drug-nutrient interactions (Townsend, Kladder, Ayele, & Mulligan, 2002). Various nomenclature exists for herbal medicines, and as such differing approaches have been adopted related to licensing, dispensing, manufacturing and trading of these products (Azaizeh, et al., 2010). Fatal adverse effects have been reported from the use of herbal products and traditional medicines resulting from contamination with microbes, excessive or banned pesticides, heavy metals, chemical toxins, and the presence of orthodox drugs.(Chan, 2003) Patient preference of traditional medicines and therapies may lead to non-adherence (Townsend et al., 2002). In recent surveys by Saad et al. (Saad, Azaizeh, Abu-Hijleh, & Said, 2006) and Azaizeh et al. (Azaizeh et al., 2010), practitioners of traditional Arab herbal medicine have very limited training and knowledge, with younger practitioners posessing even less knowledge than their predecessors. Consequently, limited access to practitioners knowledgeable of TAIM may result in self-care without professional oversight of such therapies, and adverse healthcare outcomes.

## 6. Conclusion

Despite their history and growing population, knowledge about Arab and Muslim traditional practices as it relates to health and wellness is limited. A better understanding of TAIM and its elements will enhance the ability of clinicians caring for Arab and Muslim patients to provide culturally sensitive care, as it relates to their patients’ perception of health and well-being, as well as rituals and customs pertaining to the view of healers and the value of traditional therapies. Healthcare practitioners caring for Arab and Muslim patients should be aware of the potential influence of TAIM onto healthcare practices of this population. Utilizing the TAIM model enables clinicians to anticipate and respond to healthcare practices of Arab and Muslim patients by engaging them about their use of the proposed components, and thus enhancing health provision. Given the growing interest of traditional and holistic systems of medicine in the global community, the TAIM conceptual model represents a novel addition to the literature on Arab and Muslim health practices. More research is needed to understand who provides TAIM care, professional or lay, and how the TAIM traditions are being preserved and adapted. Researchers can use this comprehensive TAIM taxonomy to delve into the respective elements, and systematically examine the theoretical and therapeutic applications.
